# Different Genetic Associations of the IgE Production among Fetus, Infancy and Childhood

**DOI:** 10.1371/journal.pone.0070362

**Published:** 2013-08-01

**Authors:** Jen-Chieh Chang, Ho-Chang Kuo, Te-Yao Hsu, Chia-Yu Ou, Chieh-An Liu, Hau Chuang, Hsiu-Mei Liang, Hurng-Wern Huang, Kuender D. Yang

**Affiliations:** 1 Institute of Biomedical Sciences, National Sun Yat-sen University, Kaohsiung, Taiwan; 2 Genomic and Proteomic Core Laboratory, Department of Medical Research, Kaohsiung Chang Gung Memorial Hospital and Chang Gung University College of Medicine, Kaohsiung, Taiwan; 3 Department of Pediatrics, Kaohsiung Chang Gung Memorial Hospital and Chang Gung University College of Medicine, Kaohsiung, Taiwan; 4 Department of Obstetrics, Kaohsiung Chang Gung Memorial Hospital and Chang Gung University College of Medicine, Kaohsiung, Taiwan; 5 Department of Pediatrics, Po-Jen Hospital, Kaohsiung, Taiwan; 6 The Department of Medical Research and Development, Show Chwan Memorial Hospital in Chang Bing, Changhua, Taiwan; 7 Institute of Clinical Medical Sciences, National Yang Ming University, Taipei City, Taiwan; South Texas Veterans Health Care System and University Health Science Center San Antonio, United States of America

## Abstract

Elevation of serum IgE levels has long been associated with allergic diseases. Many genes have been linked to IgE production, but few have been linked to the developmental aspects of genetic association with IgE production. To clarify developmental genetic association, we investigated what genes and gene-gene interactions affect IgE levels among fetus, infancy and childhood in Taiwan individuals. A birth cohort of 571 children with completion of IgE measurements from newborn to 1.5, 3, and 6 years of age was subject to genetic association analysis on the 384-customized SNPs of 159 allergy candidate genes. Fifty-three SNPs in 37 genes on innate and adaptive immunity, and stress and response were associated with IgE production. Polymorphisms of the *IL13*, and the *HLA-DPA1* and *HLA-DQA1* were, respectively, the most significantly associated with the IgE production at newborn and 6 years of age. Analyses of gene-gene interactions indentified that the combination of *NPSR1*, rs324981 TT with *FGF1*, rs2282797 CC had the highest risk (85.7%) of IgE elevation at 1.5 years of age (*P* = 1.46×10^−4^). The combination of *IL13*, *CYFIP2* and *PDE2A* was significantly associated with IgE elevation at 3 years of age (*P* = 5.98×10^−7^), and the combination of *CLEC2D*, *COLEC11* and *CCL2* was significantly associated with IgE elevation at 6 years of age (*P* = 6.65×10^−7^). Our study showed that the genetic association profiles of the IgE production among fetus, infancy and childhood are different. Genetic markers for early prediction and prevention of allergic sensitization may rely on age-based genetic association profiles.

## Introduction

The prevalence of allergic diseases has dramatically increased over the past decades, especially in children [1]. Atopic diseases such as asthma, rhinitis and eczema are among the most common chronic diseases in developed countries [2], [3]. Immunoglobulin E (IgE) plays a central role in the allergic response, and elevation of total IgE levels is considered as an indicator for atopy. Recently, evidence has demonstrated that allergy sensitization may occur in fetal life [4]. A higher antenatal IgE production reflected in the elevation of cord blood IgE (CBIgE) levels has been shown to correlate with the development of aeroallergen sensitization [5], and the later development of childhood asthma [6].

A large body of evidence shows that regulation of serum IgE levels is under strong genetic control with heritability estimates of 50–80% [7], and it has been suggested that genetic regulation of basal IgE production is independent of specific responses to allergens [8]. We have previously shown that gene influence of IgE production might begin in prenatal stage [9]. To date, only a few studies have investigated IgE production as a primary trait in children at different ages. We here postulate that certain different genes in different ages may affect IgE production for the development of allergic diseases.

IgE production and allergic diseases have a complex background, affected by innate and adaptive immunity genes [10]–[15], and other oxidative, remodeling genes as well [11], [16]–[21]. Many genetic association studies have identified more than 100 genes in 22 chromosomes associated with asthma [12], [22], [23]. Some of the genes associated with asthma were replicated in different studies, but most of them were not. This may be because different studies were performed in different populations with different genetic background, age, time and environment. There are few studies doing genetic association studies with a developmental trend from newborn, infant, toddler to childhood. This study sought allergy candidate genes and their SNPs responsible for allergic diseases, allergic reaction or inflammation for a customized 384-plex SNP array analysis of genetic association of the IgE production in children at ages of newborn, 1.5, 3 and 6 years old. Gene-gene interactions can be investigated by multifactor dimensionality reduction (MDR), a method designed to translate high-dimensional genetic data into a single dimension [24], [25]. In our previous studies on antenatal IgE production, we found that 14 genes were associated with higher CBIgE levels, and that gene-gene interactions on IgE production begin at a prenatal stage [26]. In this study, we followed the same cohort to 6 years of age, and studied whether gene-gene interactions on IgE production are different among childhood from newborn to 1.5, 3, and 6 years of age. Clarification of the gene-gene interactions on IgE production among fetus, infancy and childhood stages may allow us to tract the development of allergic sensitization, and render the prediction and prevention of allergic diseases at the perinatal stage.

## Materials and Methods

### Subjects and Blood Sample Collection

To study the association between genetic polymorphisms and blood IgE levels, we collected blood samples from neonatal umbilical cord blood in a birth cohort study after informed consent as previously described [27], [28]. Of the 571 in the cohort study [26], 469 infants at 1.5 years, 491 at 3 years, and 409 at 6 years of age completed the follow-up. The study protocol was approved by the Institutional Review Board of the Chang Gung Memorial Hospital, Taiwan. Because this is a birth cohort follow-up study, the initially informed consent was obtained from parents in prenatal stage, and the informed re-consent was obtained in each session of questionnaire. All parents signed the consent in written form after the informed communications.

### IgE Measurements and DNA Extraction

Total IgE levels were measured using a Pharmacia CAP system (Pharmacia & Upjohn Diagnostics AB, Uppsala, Sweden). We used Pharmacia Cap System to detect allergic sensitization as reflected on allergen specific IgE levels. The allergen specific IgE levels detected by Pharmacia Cap System have been shown to closely correlate to skin prick tests [29]–[31]. In consideration of skin reactivity to allergen challenge in young children is lower and the multiple skin pricks in different age of 1.5, 3 and 6 years are painful to small children. This study used in vitro specific IgE detection by the Pharmacia Cap System. Total IgE measurements were measured at ages newborn, 1.5, 3 and 6 years and analyzed in tertiles. Blood DNA samples were extracted by 0.5% SDS lysing buffer followed by proteinase K (1 mg/ml) digestion, phenol-chloroform extraction, and 70% alcohol precipitation as our previous description [27], [28], [32].

### Amplification of Genomic DNA for Oligonucleotide-based 384 SNP Multiplex Microarray Assay and Verification

In an attempt to select 384 customized SNPs in and around allergy candidate genes for this study, we searched allergy candidate genes and its SNPs associated with IgE production or allergic diseases reported in literature [10]–[15], [26], and also included the genes whose SNP(s) was (were) associated with immune responses. Moreover, we included oxidative stress and remodeling genes in this study because certain oxidative stress response and remodeling genes such as *ADAM33*, *CAT*, *NOS1*, *NOS2*, *NOS3*, *GSTP1* and *MSRA* have been shown to be responsible for modulation of allergy reaction in prenatal or postnatal environment [11], [16]–[21]. These SNPs were preliminarily screened via a proprietary algorithm of Illumina (San Diego, CA, USA) that predicts the performance of oligonucleotide hybridization on the Illumina platform. After excluding the SNPs with minor allele frequency <0.05 in Chinese population, we selected 91 SNPs in 44 innate genes, 102 SNPs in 37 adaptive genes, and 191 SNPs in 78 oxidative stress and remodeling genes for the 384 customized SNPs design (Fig. S1), showing all the genes selected on different chromosomes. The 384 SNPs in 159 allergy candidate genes with representative SNPs in the NCBI Genome Build 36.3 (dbSNP build 129) were genotyped with an Illumina BeadStation 500 GX.

In brief, DNA samples were quantified with a PicoGreen dsDNA Quantitation Kit (Molecular Probes, Eugene, OR, USA). After DNA quantification, the DNA samples were adjusted to 50 ng/ul in TE buffer (Tris–HCl, 10 mM, EDTA 1 mM, pH 8.0) for PCR amplification in an Oligo Pool All (OPA) containing a set of all the primers for the 384 SNPs. The PCR products were hybridized and analyzed using BeadStudio software with genotyping analysis module version 2.3.43 (Illumina, San Diego, CA, USA). The overall call rate of the 384 SNPs was 97.1%. After excluding 14 SNPs with a call rate less than 90%, the average call rate of the SNPs subjected to the analysis was 99.0%. Verification of the accuracy of the 384 SNP microarray data by repeat measurements and parallel experiments of restriction fragment length polymorphisms of *IL13*, 2044 A/G and *CTLA-4*, 49 A/G was performed as described in our previous report [26]. The genotyping accuracy for the *IL13*, 2044 A/G polymorphism between these two methods was 99%, and that for the *CTLA-4*, 49 A/G polymorphism between these two methods was 98%.

### Data Analysis and Statistics

The associations of elevated IgE in subjects with different genotypes or alleles were analyzed by univariate analyses using SPSS 17.0 (SPSS, Inc., Chicago, IL, USA). Genes associated with IgE elevation were arbitrarily classified into three categories: innate immunity, adaptive immunity, and stress and response genes. Total IgE levels are not normally distributed. We followed previous articles to use quartile classification [33], [34] for the Chi-square test of risk SNPs to higher IgE production in children with different ages of 1.5, 3 and 6 years old in this study (higher vs. lower tertiles). Cord blood IgE levels were presented and analyzed as in our previous description [26]. IgE levels at the ages of 1.5, 3, and 6 years were cut off at <25% and >75% to define the high and low IgE groups for statistical analyses. The intra-correlations of the IgE levels among newborn, 1.5, 3, and 6 years of age were analyzed by nonparametric correlation.

Gene-gene interactions were analyzed by multifactor dimensionality reduction software (MDR version 1.1.0), which is an open source project available at http://sourceforge.net/projects/mdr/. The MDR approach of the gene-gene interactions has been described previously [24]–[26]. The MDR method includes a combined cross-validation/permutation testing procedure that minimizes the false-positive findings that may otherwise result from multiple comparisons. In brief, cross-validation consistency (CVC) and prediction error were calculated for each combination of a pool of polymorphisms. The model with the combination of items that maximizes the CVC and minimizes the prediction error is selected to be the best model. The statistical significance by comparing the average prediction error from the observed data with the distribution of average prediction errors under the null hypothesis of no associations derived empirically from 10,000 permutations. The null hypothesis was rejected when the *P* value derived from the permutation test was 0.05 or less. Because IgE elevation was defined by a binary cut-off at >75% vs. <25% rather than continuous variable in this study, we used a binary logistic regression to confirm the high and low risk classifications of the significant gene-gene interactions revealed by MDR analyses. Moreover, we also used rate displays of the IgE elevation for Chi-square tests to validate the differences between high and low risk genotypes using the whole dataset at the ages of 1.5, 3 and 6 years.

## Results

### Subjects and Demographic Data

Of the 571 DNA samples with genotyping of the 384 SNPs in the 159 allergy candidate genes (Fig. S1) in our cohort study [26], 469, 491 and 409 subjects completed follow-up at 1.5, 3 and 6 years of age, respectively. The demographic data of the subjects at 1.5, 3 and 6 ages were comparable with those of the 571 subjects with cord blood (CB) samples studied in our previous report [26]. There were no significant differences in gender, family atopy history, parental smoking history, prematurity or CBIgE elevation. The mean IgE levels (± standard deviation) at newborn, 1.5, 3 and 6 years of age were, respectively, 0.36±1.34, 120.70±212.31, 152.29±265.73 and 223.20±409.16 kU/L. The characteristics of the infants participating in this genetic study are presented in Table 1. The demographic data among subjects studied at newborn, 1.5, 3 and 6 years of age were not significantly different (*P*>0.05). The intra-correlations of IgE levels among newborn, 1.5, 3 and 6 years were highly significant correlated one another (*P*<10^−6^; Table 2). The correlations of CBIgE levels to the total IgE levels in childhood were significantly correlated (r = 0.25–0.27), but lower than the correlations of the IgE levels among 1.5, 3, and 6 years of age (r = 0.59–0.75). The highly significant intra-correlations of IgE levels among different ages (newborn, 1.5, 3 and 6 years of age) suggest that allergic sensitization with IgE production occurs early in perinatal stage. Moreover, we found that total IgE levels in the Taiwanese children were higher than those in children of other cohorts [33], [35].

**Table 1:** Demographic data of the cohort population from newborn to 6 years of age.

Demographic data	Newborn* (n = 571)	1.5 y/o (n = 469)	3 y/o (n = 491)	6 y/o (n = 409)	*p* values
Median total IgE levels (kU/L) (25th, 75th percentile)	0 (0, 0.41)	49.20 (21.85, 140.00)	61.20 (22.90, 143.00)	69.40 (22.60, 245.00)	–
Maternal atopy#	24.50%	24.30%	24.20%	23.00%	0.951
Paternal atopy#	25.00%	25.20%	26.50%	26.20%	0.940
Prematurity†	5.10%	5.50%	5.70%	5.10%	0.965
Gender (Male)	53.40%	55.20%	55.00%	53.80%	0.924
Parental smoking	34.60%	34.10%	34.80%	34.50%	0.996
CB IgE (≥0.5 kU/L)	21.50%	19.60%	21.60%	20.80%	0.861

* A higher total IgE level in cord blood (newborn) was cut-off at ≥0.5 kU/L as previously described; higher total IgE levels at 1.5, 3 and 6 years of age were defined as above the 75th percentile of the total IgE collected from the study population.

# Atopy was defined as phenotypic asthma, rhinitis and/or atopic dermatitis along with detectable serum specific IgE to one or more common allergens.

† Prematurity was defined as a gestational age <37 weeks. Demographic data among the different age groups were not significantly different (Chi-square test).

**Table 2:** The intra-correlations of the IgE levels among newborn, 1.5, 3 and 6 years of age (y/o).

	1.5 y/o	3 y/o	6 y/o
Newborn	0.262	0.252	0.259
1.5 y/o		0.747	0.590
3 y/o			0.739

Data presented are correlation coefficient. All *P* values are <10^−6^ by Spearman’s correlation.

### Associations of Gene Polymorphisms with IgE Production during Childhood

Of the 384 SNPs studied, a total of 350 SNPs were included for the final analysis after excluding those with a call rate less than 90% or beyond the Hardy-Weinberg equilibrium (HWE) [26]. We used the Chi-square test to analyze whether certain genotypes were associated with elevation of IgE levels at ages 1.5, 3 and 6 years (higher (>75th) vs. lower (<25th) percentile), tested the gene-gene interactions by MDR analyses and validated the risk gene-gene interactions on IgE production by the rate displays of Chi-square tests.

**Figure 1:**
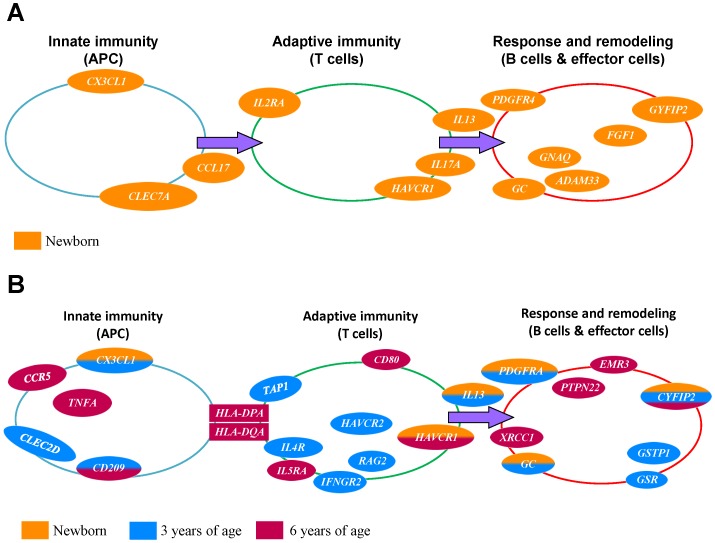
37 genes were associated with IgE elevation in different ages. Genes in innate immunity, adaptive immunity, and stress and response associated with IgE production from newborn (CB) to childhood at 3 and 6 years of age. Genes associated with CBIgE elevation are indicated in orange (A). Genes associated with IgE elevation persistent into childhood are indicated in orange, blue and/or red color. Those associated with IgE elevation at 3 years of age is labeled in blue, and those at 6 years of age are labeled in red (B).

We have previously shown that twenty-one SNPs in 14 genes are associated with CBIgE elevation (≧ 0.5 kU/L) [26] (summarized as Table 3 and Fig. 1A). Here, we found that 53 SNPs in 37 genes were associated with IgE elevation in different ages (newborn, 1.5, 3 and 6 years) using univariate analysis (UVA). The *IL-13*, rs1800925 was the most significantly associated with CBIgE elevation, and *HLA-DPA1*, rs1431399 was the most significantly associated with the IgE elevation at 6 years of age. Five genes associated with CBIgE elevation persisted into childhood associated with infant IgE elevation at 3 years of age. The *CD209* polymorphisms were associated with infant IgE elevation both at 3 and 6 years of age. The *CYFIP2* polymorphisms were associated with IgE elevation at newborn, 3 and 6 years of age (Table 3; Fig. 1B).

**Table 3:** Association of 53 SNPs in 37 innate immunity, adaptive immunity, and stress and response genes with total IgE elevation.

Genes	dbSNP BUILD 129	Protective genotype	Risk genotype	Newborn*	1.5 y/o	3 y/o	6 y/o
Innate immunity							
*CCR5*	rs1799987	AA, AG	GG	ns	ns	ns	0.009
*TNFA*	rs1800629	GG	AG	ns	ns	ns	0.019
*IL6*	rs1880242	GG	TT, TG	ns	0.008	ns	ns
*CLEC2D*	rs1560011	AA	AG	ns	ns	0.043	ns
*CLEC7A*	rs2078178	CC	TT, TC	0.048	ns	ns	ns
*CLEC9A*	rs874463	AT	TT	ns	0.046	ns	ns
*CCL17*	rs223895	CC	TC	0.004	ns	ns	ns
*CCL17*	rs223900	CC	TC	0.001	ns	ns	ns
*CX3CL1*	rs170361	AA^a^, GG^a,b,c^	AG	0.004	0.034	0.043	ns
*CX3CL1*	rs4151117	TT, GG	TG	2.39×10^−4^	ns	ns	ns
*CX3CL1*	rs8323	AA	AG	0.014	0.021	ns	ns
*CD209*	rs4804801	AA, AT	TT	ns	ns	0.012	ns
*CD209*	rs7248637	AG	GG	ns	ns	ns	0.05
Adaptive immunity							
*IL5RA*	rs163550	CC, CG	GG	ns	ns	ns	0.043
*IL5RA*	rs340833	GG	AG	ns	ns	ns	0.03
*CD80*	rs2629396	AC, CC	AA	ns	ns	ns	0.003
*IL13*	rs1295686	GG	AA, AG	1.60×10^−4^	ns	0.012	ns
*IL13*	rs1800925	CC	TC	2.62×10^−4^	ns	0.002	ns
*IL13*	rs20541	GG	AA^a^, AG^a,c^	1.86×10^−4^	ns	0.007	ns
*IL9*	rs10477776	AG	GG	ns	0.041	ns	ns
*HAVCR1*	rs953569	AA^a^, AC^d^	AC^a^, AA^d^	0.015	ns	ns	0.04
*HAVCR2*	rs11134551	AG	AA	ns	ns	0.049	ns
*IL17A*	rs3748067	AG, GG	AA	0.024	ns	ns	ns
*HLA-DPA1*	rs1431399	AG, GG	AA	ns	ns	ns	1.87×10^−4^
*HLA-DQA1*	rs2040410	GG	AG	ns	ns	ns	0.004
*TAP1*	rs2284190	TC	TT	ns	0.009	0.037	ns
*IL2RA*	rs4749926	GG	AA	0.044	ns	ns	ns
*RAG2*	rs867801	TC	TT	ns	0.003	0.01	ns
*IL4R*	rs1801275	AA	AG	ns	ns	0.05	ns
*IFNGR2*	rs2268241	AA	AG, GG	ns	0.023	0.016	ns
*IFNGR2*	rs9808753	GG	AA, AG	ns	0.011	0.017	ns
*IFNGR2*	rs9976971	GG	AA, AG	ns	ns	0.032	ns
Stress and response							
*PTPN22*	rs3765598	TC	CC	ns	ns	ns	0.013
*PDGFRA*	rs4358459	TT^a,c^, TG^a^	GG	0.041	ns	0.028	ns
*GC*	rs222016	AA	AG	0.031	ns	ns	ns
*GC*	rs4588	AA	AC, CC	ns	ns	0.046	ns
*FGF1*	rs2278688	CG, GG	CC	0.023	ns	ns	ns
*FGF1*	rs2282797	TT, TC	CC	0.022	0.026	ns	ns
*CYFIP2*	rs2863198	CC	CG	ns	ns	ns	0.006
*CYFIP2*	rs3734028	AA^a^, GG^d^	AG	0.046	ns	ns	0.012
*CYFIP2*	rs767007	GG	CG	ns	ns	0.045	0.01
*GSR*	rs8190955	CC	TC	ns	ns	0.006	ns
*GNAQ*	rs4744850	CC^a^, CG^a,b^	GG	0.02	0.044	ns	ns
*C11orf74*	rs2422297	CG	GG	ns	0.001	0.009	ns
*EHF*	rs717582	AG, GG	AA	ns	0.024	ns	ns
*C11orf72*	rs12790798	AA	AG	0.003	ns	ns	ns
*GSTP1*	rs1871042	CC	TC	ns	ns	0.009	ns
*EMR3*	rs10410565	CG	CC	ns	ns	ns	0.027
*XRCC1*	rs25487	AA	AG	ns	ns	ns	0.044
*ADAM33*	rs2280091	AA	AG	ns	0.008	ns	ns
*ADAM33*	rs3918395	GG	TG	ns	0.019	ns	ns
*ADAM33*	rs528557	CC	CG	0.041	ns	ns	ns
*ADAM33*	rs612709	GG	AG	0.05	ns	ns	ns

ns, not significant. a, newborn; b, 1.5 y/o; c, 3 y/o; d, 6 y/o.

* The results were derived from our previous study [26].

While we arbitrarily clustered whether the genes associated with IgE production are related to innate immunity, adaptive immunity or stress and response genes, we found that 13 SNPs in 9 innate immunity genes conferred risk for or protected against elevated IgE levels. These innate immunity genes, namely *CCR5*, *TNFA*, *IL6*, *CLEC2D*, *CLEC7A*, *CLEC9A*, *CCL17*, *CX3CL1* and *CD209*, were significantly associated with higher or lower IgE levels. These genes were mainly clustered in chromosomes 3, 6, 7, 12, 16 and 19 (Table 3). Nineteen SNPs in 14 adaptive immunity genes were associated with higher or lower IgE levels. These adaptive immunity genes were *IL5RA*, *CD80*, *IL13*, *IL9*, *HAVCR1*, *HAVCR2*, *IL17A*, *HLA-DPA*, *HLA-DQA*, *TAP1*, *IL2RA*, *RAG2*, *IL4R* and *IFNGR2*, mainly clustered in chromosomes 3, 5, 6, 10, 11, 16 and 21 (Table 3). We also discovered 21 SNPs in 14 stress and response genes associated with higher or lower IgE levels at newborn, 1.5, 3 and 6 years of age. These stress and response genes, including *PTPN22*, *PDGFRA*, *GC*, *FGF1*, *CYFIP2*, *GSR*, *GNAQ*, *C11orf74*, *EHF*, *C11orf72*, *GSTP1*, *EMR3*, *XRCC1*, and *ADAM33,* were mainly clustered in chromosomes 1, 4, 5, 8, 9, 11, 19 and 20 (Table 3). We formulated these genes associated with IgE production from newborn, 3, and 6 years of age in an immune response pathway scheme as shown in Fig. 1. *CX3CL1*, *IL13*, *IL17A*, *PDGFRA*, *ADAM33*, etc. were associated with the CBIgE elevation; *CX3CL1*, *CYFIP2*, *PDGFRA*, *GC*, and *IL13* were associated with the IgE elevation at newborn and 3 years of age; *HLA-DPA1*, *HLA-DQA1*, *CCR5*, *IL5RA*, *CD80*, etc. were associated with the IgE production at 6 years of age.

### Gene-gene Interactions on IgE Production during Childhood by MDR Analysis

Given the important role of genes related to higher or lower IgE production, we sought to study gene-gene interactions on IgE production at newborn, 1.5, 3 and 6 years of age. Multifactor dimensionality reduction analysis was used to investigate whether certain SNP combinations of the 350 SNPs studied were significantly associated with IgE production (Table 4). In MDR, we found significant combinations on two-way and three-way models of gene-gene interactions, but not on four-way model of IgE production at newborn, 1.5, 3, and 6 years of age (Table 4). The combination of *NPSR1*, rs324981 and *FGF1*, rs2282797 was associated with IgE production at 1.5 years of age; the combination of *IL13*, rs1800925, *CYFIP2*, rs767007 and *PDE2A*, rs755933 was associated with IgE production at 3 years of age; and the combination of *CLEC2D*, rs1560011, *COLEC11*, rs10210631 and *CCL2*, rs2857656 was associated with IgE levels at 6 years of age. The false positive rate (FPR) and false negative rate (FNR) of these putative combinations were 0.385, 0.258 and 0.350 (FPR); 0.299, 0.341 and 0.225 (FNR), respectively, at 1.5, 3, and 6 years of age.

**Table 4:** Summary of the gene-gene interactions on total IgE production among newborns, infancy and early childhood.

Interactions*	Best Model		Average testing Bal. Acc. (%)	Average CV consistency	*P* value§
	**Total CBIgE** †				
1	*IL13* (rs1295686)		55.1	11/20	0.060
2	*ADAM33* (rs597980)	*CX3CL1* (rs4151117)	51.9	6/20	0.252
3	*CCL17* (rs223900)	*PTPN22* (rs1217395)	55.5	13/20	0.006
	*CX3CL10* (rs867562)				
4	*CCL17* (rs223900)	*IL5RA* (rs163550)	48.1	10/20	0.748
	*CLECL1* (rs741199)	*LY75* (rs1365801)			
	**Total IgE** # **, 1.5 years of age**				
1	*ADAM33* (rs528557)		47.4	6/20	0.868
2	*NPSR1* (rs324981)	*FGF1* (rs2282797)	62.4	19/20	0.006
3	*CD14* (rs2569190)	*TXN* (rs4135218)	54.3	9/20	0.058
	*MSRA* (rs6984840)				
4	*ADAM33* (rs528557)	*CD14* (rs2569190)	51.8	11/20	0.132
	*TXN* (rs4135218)	*CLECL1* (rs2268146)			
	**Total IgE** # **, 3 years of age**				
1	*IL13* (rs1800925)		57.4	18/20	0.021
2	*IL13* (rs1800925)	*CXCL12* (rs2297630)	49.4	8/20	0.588
3	*IL13* (rs1800925)	*CYFIP2* (rs767007)	63.1	20/20	<0.001
	*PDE2A* (rs755933)				
4	*CD14* (rs2569190)	*IFNGR2* (rs2268241)	53.4	14/20	0.058
	*GPIAP1* (rs7113413)	*CAT* (rs566979)			
	**Total IgE** # **, 6 years of age**				
1	*CD80* (rs2629396)		47.3	10/20	0.132
2	*PTPN22* (rs3765598)	*IL5RA* (rs163550)	42.5	7/20	0.979
3	*CLEC2D* (rs1560011)	*CCL2* (rs2857656)	67.2	19/20	<0.001
	*COLEC11* (rs10210631)				
4	*NPSR1* (rs323922)	*IL2RA* (rs3118470)	45.1	5/20	0.588
	*CLEC2D* (rs1560011)	*CCL2* (rs2857656)			

CV, Cross-validation; Bal. Acc., Balance accuracy.

* Number of loci considered.

§ Significance of prediction error (empirical *P* value based on 10,000 permutations).

† Cut off ≥0.5 (kU/L).

# Higher (>75%) vs. lower tertile (<25%).

**Figure 2:**
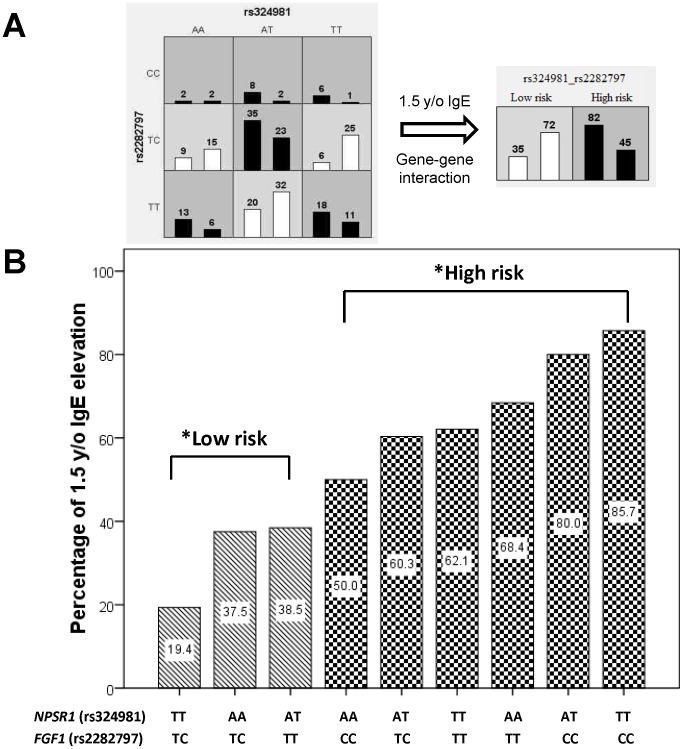
Gene-gene interaction on IgE elevation at 1.5 years of age. The display and validation of the gene-gene interactions on IgE elevation between *NPSR1*, rs324981 and *FGF1*, rs2282797. The left column in all boxes represented the case number whose IgE levels were higher than 75th percentile, and the right column in all boxes showed their IgE levels were lower than 25th percentile. The high-risk genotypes with an increase in total IgE levels were defined as the ratio of left to right column greater than or equal to the threshold of 1.0, whereas low-risk groups were defined as the threshold were less than 1.0. The nine combinations of different genotypes were classified into high (black bars) and low (white bars) risk groups of IgE production by MDR analysis (A). The differences between high and low risk classifications were validated by the Chi-square test (^*^ *P*<0.001), showing that the combination of *NPSR1*, rs324981 TT with *FGF1*, rs2282797 CC had the highest risk of infant IgE elevation at 85.7%, and the combination with *NPSR1*, rs324981 TT and *FGF1*, rs2282797 TC had the lowest risk of infant IgE elevation at 1.5 years of age of 19.4% (B).

To validate the gene-gene interactions revealed by MDR analyses, we controlled for the significant SNPs of allergy candidate genes at the ages of 1.5, 3 and 6 years and for the perinatal factors including maternal atopy, paternal atopy, parental smoking, gender, and maturity in the multivariate analysis. As shown in Table S1A, the interaction of *NPSR1*, rs324981 and *FGF1*, rs2282797 was significantly associated with IgE production at 1.5 years of age (*P = *1.46×10^−4^). The interaction among *IL13*, rs1800925, *CYFIP2*, rs767007 and *PDE2A*, rs755933 was significantly associated with IgE production at 3 years of age (Table S1B, *P = *5.98×10^−7^). The interaction among *CLEC2D*, rs1560011, *COLEC11*, rs10210631 and *CCL2*, rs2857656 was significantly associated with IgE levels at 6 years of age (Table S1C, *P = *6.65×10^−7^). We also validated the risk classifications by rate displays of the Chi-square test. As shown in Fig. 2A, we evaluated the two-way model of MDR on the interaction between *NPSR1*, rs324981 and *FGF1*, rs2282797 and then validated the significantly different (*P*<0.001) high and low risk classifications by the Chi-square test (Fig. 2B). In the nine interaction items, the combination of *NPSR1*, rs324981 TT with *FGF1*, rs2282797 CC had the highest risk (85.7%) of IgE production at 1.5 years of age, and the combination with *NPSR1*, rs324981 TT and *FGF1*, rs2282797 TC had the lowest risk (19.4%) of infant IgE production at 1.5 years of age.

In the three-way model of gene-gene interactions, the combination of *IL13*, rs1800925, *CYFIP2*, rs767007 and *PDE2A*, rs755933 had the highest average testing balance accuracy at 63.1%, classifying high and low risk classifications to the IgE elevation at 3 years of age (Fig. S2A). The differences between the high and low risk classifications were validated by the rate displays of the Chi-square test on IgE production at 3 years of age (Fig. S2B), showing the highest risk (100%) of IgE elevation at 3 years of age in the combination of three *IL13, CYFIP2*, and *PDE2A* genotypes. Similarly, the three-way model of gene-gene interactions on elevation of IgE levels at 6 years of age were classified into high and low risk groups by MDR (Fig. S3A), and validated by rate displays of IgE elevation on the Chi-square test. The combination of *CLEC2D*, rs1560011 AG, *COLEC11*, rs10210631 AA, and *CCL2*, rs2857656 GC or the combination of *CLEC2D*, rs1560011 GG, *COLEC11*, rs10210631 AA, and *CCL2*, rs2857656 CC had the highest risk for higher IgE production (Fig. S3B). Fig. S4 showed longitudinal analyses of the trajectory of IgE production from newborn to 6 years of age based on the best MDR models of gene-gene interactions for the IgE production at different ages. The higher risk classifications of gene-gene interactions for CBIgE elevation identified by MDR analysis were significantly associated with the higher IgE production at 1.5 and 3 years of age but not at 6 years of age (Fig. S4A). The higher risk classifications of gene-gene interactions for the higher (>75%) IgE production at 1.5 years of age identified by MDR analysis were significantly associated with the higher IgE production at all age groups (newborn, 1.5, 3 and 6 years of age; Fig. S4B). The higher risk classifications of gene-gene interactions for the higher IgE production at 3 years of age were significantly associated with the higher IgE production at 1.5 and 6 years of age (Fig. S4C). The higher risk classifications of gene-gene interactions for the higher IgE production at 6 years of age were significantly associated with the higher IgE production in all age groups (Fig. S4D). This suggests that the genetic association with CBIgE production could predict the IgE production until 3 years of age, and the genetic association with IgE production at 1.5 years of age could predict IgE production at 3 and 6 years of age.

## Discussion

Understanding the genes susceptible to higher IgE production may provide a way to probe the functional genetics of IgE-mediated allergic diseases [10]. Data accumulated indicate that a distinct subphenotype with elevated IgE levels has a discrete genetic control independent of other subphenotypes involved in atopic diseases [36]. Many different genes or loci have been associated with IgE production and/or asthma in different studies [10], [12], [13], [15], [26]–[28], [32]. However, not many allergy candidate genes could be replicated or validated in different studies [10], [12], [13], [15], [26]–[28], [32]. A review article summarizing dozens of genome-wise association study (GWAS) for asthma and allergy related traits has shown that few genetic variants give the highest risk to allergy related traits but limited to certain populations indicating a complex origin of allergic diseases [37]. Recently, a GWAS study focusing on childhood asthma has identified a locus on chromosome 17q12-21 (encoding ORMDL3 and GSDMB) as a risk factor for predominantly childhood-onset asthma, but not for atopy, and overall not for adult-onset asthma [38]. The wide variations of the genes associated with IgE-mediated diseases may in part be explained by different polymorphisms in different populations, different environments and the statistical methods used to detect gene-gene interactions. The GWAS was not made in this study because it is too expensive for us to perform 571 cohort samples using GWAS approach. Thus, we sorted allergy candidate genes and immune related genes for customized chips with 384 SNPs. Employing the 384-customized SNPs, we studied and demonstrated the kinetic associations of IgE production with different gene profiles at newborn, 1.5, 3 and 6 years of age. The polymorphism of IL-13 was the most significantly associated with CBIgE production, and the HLA class II alleles were not associated with IgE production until children at 6 years of age.

The HLA genotype dictates an individual’s repertoire of the peptide presentation to lymphocytes [39]. Studies of HLA Class II alleles have established that the locus inﬂuences total and/or specific IgE levels [40]–[45]. Cookson et al. [44], [46] showed that HLA-DRB1 is responsible for a greater proportion of the variation in total IgE levels in different ethnic populations. A recent GWAS from the Framingham cohort [41] found that *HLA-G*, *HLA-A* and *HLA-DQA2* were associated with total serum IgE levels. Another GWAS from the United States also demonstrated that *HLA-DR/DQ* region on chromosome 6p21 was associated with asthma [47]. It is well-known that HLA molecules are highly polymorphic in different races and populations. However, there is little theoretical basis upon what to assume that HLA alleles are associated with IgE production in different ages. In this study, we found that the SNPs of *HLA-DPA1* and *HLA-DQA1*, although not associated with IgE production in children under 3 years of age, were significantly associated with total IgE production when children reached 6 years of age. IgE production associated with innate immunity in earlier ages, but with HLA genotypes at 6-year old suggests that infants and small children may develop allergic sensitization depending on the interactions between certain genetic polymorphism and environmental exposure in different ages. Since children 6 years of age have greater exposure to a wider range of antigens than those under 3 years of age, further studies to validate whether *HLA-DPA1* and *HLA-DQA1* interact specifically with environmental factors for antigen presentation and allergic responses are warranted.

Interestingly, we also found that the SNPs of *IL13* and *CX3CL1* were the most significantly associated with IgE production in newborns at *P* values around 10^−4^, whose *P* value decreased to around 10^−2^ to 10^−3^ at 1.5 and 3 years of age, respectively, and down to no significant difference (*P*>0.05) at 6 years of age. In contrast, the SNPs of *HLA-DPA1* and *HLA-DQA1* revealed a significant association with IgE production when the children grew up to 6 years of age. IgE is produced by B cells, which interact with T_H_2 cells and undergo isotype class-switching after the induction of T_H_2 cell-derived cytokines. It is well known that an imbalance between T_H_1 and T_H_2 immune response is critical for IgE production and to the subsequent development of allergic diseases. In addition, evidence has suggested that inappropriate T_H_1 and T_H_2 responses can be suppressed by regulatory T (Treg) cells [48]. IL-13 is an important T_H_2 cytokine, which increased in the sera of individuals with atopic diseases and also in the bronchoalveolar lavage fluids of asthmatic patients. The overexpression of IL-13 in the airway leads to an increase in mucus production, bronchial hyperresponsiveness, and goblet cell hyperplasia [49], [50]. Additionally, it had been suggested that IL-13 might inhibit the development of arthritis in animal models, and that an increase in production of IL-13 significantly correlates with a reduction in pro-inflammatory cytokines. A humanized IL-13 monoclonal antibody, lebrikizumab displayed pharmacodynamic activity in reducing serum total IgE levels [51]. These do support the role of IL-13 in regulating IgE levels. The genetic association of *IL13* with CBIgE production has been reported in our birth cohort [26] and two other groups [52], [53]. In this study, we reported its association with IgE production persists from newborn stage to 3 years of age. The different combinations of *IL13, CYFIP2*, and *PDE2A* genotypes caused different high and low risk classifications for IgE production at 3 years of age, showing the highest risk in the combinations of *IL13*, rs1800925 CC, *CYFIP2*, rs767007 CC and *PDE2A*, rs755933 AA or *IL13*, rs1800925 TC, *CYFIP2*, rs767007 CC and *PDE2A*, rs755933 AG or *IL13*, rs1800925 TC, *CYFIP2*, rs767007 GG and *PDE2A*, rs755933 AA. Different gene-gene interactions associated with IgE production among newborn, infancy and childhood suggests prediction of allergic sensitization in perinatal stage may rely on age-based genetic association profiles, and environmental factors in different ages.

In this study, we initially analyzed associations of the 350 SNPs with IgE elevation and arbitrarily summarized the risk SNPs into different compartments of the immune genes studied. We then traced kinetic changes of the genetic associations from newborn, 1.5, 3 to 6 years of age. For gene-gene interactions are difficult to be assessed by multivariate logistic regression analyses, we used MDR analysis, which could identify high order gene-gene interactions without previous information of disease-associated genes [54], [55], to detect gene-gene interactions based on the cross-validation/permutation testing of the 350 SNPs dataset matched to rates of the IgE elevation in different ages of children. We found that the higher risk classifications of gene-gene interactions on the CBIgE elevation identified by MDR analysis were longitudinally associated with higher (>75th percentile) IgE production until 3 years of age. The higher risk classifications of gene-gene interactions on the higher IgE production at 1.5 years of age identified by MDR analysis were significantly correlated to the higher IgE production at all age groups (newborn, 1.5, 3 or 6 years of age). The results are compatible to the intra-correlations of the IgE production among 0, 1.5, 3 and 6 years of age by Spearman’s correlation (Table 2), in which the higher IgE production at 1.5 years of age was more significantly than the higher CBIgE levels on the association with the IgE production at 3 or 6 years of age. This suggests that MDR is a suitable method to identify gene-gene interactions on the higher IgE production, even in the analysis of genetic associations with longitudinal trajectory of IgE production from birth, 1.5, 3 and 6 years of age.

The CBIgE levels detected in this cohort are similar to other cohorts in Germany and Canada [35], [56], [57]. However, when we continued to follow up the total IgE levels in this Taiwanese cohort, their total IgE levels in childhood were much higher than other prospective follow-up groups in the literature [33], [35]. Hence, different exposures interact with various genetic backgrounds in a range of age, racial or ethnic groups as well as socioeconomic status will eventually result in different concentrations of IgE production and IgE-mediated diseases. Prevention of IgE production and IgE-mediated diseases may be made possible by control of different environments for the subjects with susceptible genotypes early in the perinatal stage. Further studies to compare gene-gene and gene-environment interactions on IgE production in different ethnic populations are necessary.

The strengths of this study are its larger sample size and prospective follow-up to 6 years of age, which enabled us to evaluate the kinetic genetic associations of IgE production during early childhood. The limitations of the study are its analysis with dichotomical dataset to do permutation; the dichotomical cut-off values of IgE levels at 75th vs. 25th percentile may not completely represent the high and low risk groups. This study only analyzed serum IgE pool because this study did not measure cell bound IgE levels that are age-dependent [58]. The cell bound IgE may affect the total IgE in blood and influence the associations between genes and total IgE levels. These limitations should be considered in future studies.

## Supporting Information

Figure S1 Candidate genes on list on chromosomes. A list of the customized 384 SNPs in 159 allergy candidate genes on 22 somatic and X chromosomes. (TIFF)

Figure S2 Gene-gene interaction on IgE elevation at 3 years of age. The display and validation of the gene-gene interactions among *IL13, CYFIP2*, and *PDE2A* genes. Twenty combinations of different genotypes were classified into high (black bars) and low (white bars) risk groups of IgE production by MDR analysis (A). The differences between high and low risk classifications for IgE production at 3 years of age were validated by the Chi-square test (^*^ *P*<0.001), showing the highest risk (100%) of infant IgE elevation at 3 years of age in the combination of three *IL13, CYFIP2*, and *PDE2A* genotypes (B). (TIFF)

Figure S3 Gene-gene interaction on IgE elevation at 6 years of age. The display and validation of the gene-gene interactions among *CLEC2D, COLEC11*, and *CCL2* genes. Twenty-four combinations of different genotypes were classified into high (black bars) and low (white bars) risk groups of IgE production by MDR analysis (A). The differences between high and low risk classifications for IgE production at 6 years of age were validated by the Chi-square test (^*^ *P*<0.001), showing the highest risk (100%) of infant IgE elevation at 6 years of age in the combination of two *CLEC2D, COLEC11*, and *CCL2* genotypes (B). (TIF)

Figure S4 MDR analyses of longitudinal trajectory of IgE production from newborn to 6 years of age. The combination of *CCL17*, rs223900, *PTPN22*, rs1217395 and *CX3CL10*, rs867562 significantly associated with CBIgE production at newborn stage retained its association with the higher IgE production at 1.5 and 3 of ages, but not 6 years of age (A). The combination of *NPSR1*, rs324981 and *FGF1*, rs2282797 associated with the higher IgE production at 1.5 years of age was significantly associated with the higher IgE production at all age groups (birth, 3 and 6 years of age) (B). The combination of *IL13*, rs1800925, *CYFIP2*, rs767007 and *PDE2A*, rs755933 associated with the IgE production at 3 years of age was significantly associated with the higher IgE production at 1.5 and 6 years of age (C). The combination of *CLEC2D*, rs1560011, *COLEC11*, rs10210631 and *CCL2*, rs2857656 associated with the higher IgE production at 6 years of age was significantly associated with the higher IgE production at all age groups (birth, 1.5 and 3 years of age) (D). (TIF)

Table S1 A. Validation of the gene-gene interactions by MDR analysis on IgE production at 1.5 years of age in multivariate logistic regression. B. Validation of the gene-gene interactions by MDR analysis on IgE production at 3 years of age in multivariate logistic regression. C. Validation of the gene-gene interactions by MDR analysis on IgE production at 6 years of age in multivariate logistic regression. (DOC)
