# Supplementary figures and images for: Different Genetic Associations of the IgE Production among Fetus, Infancy and Childhood
Source: PLoS One. 2013 Aug 1;8(8):e70362. doi: 10.1371/journal.pone.0070362 (PMC3731352; doi:10.1371/journal.pone.0070362)

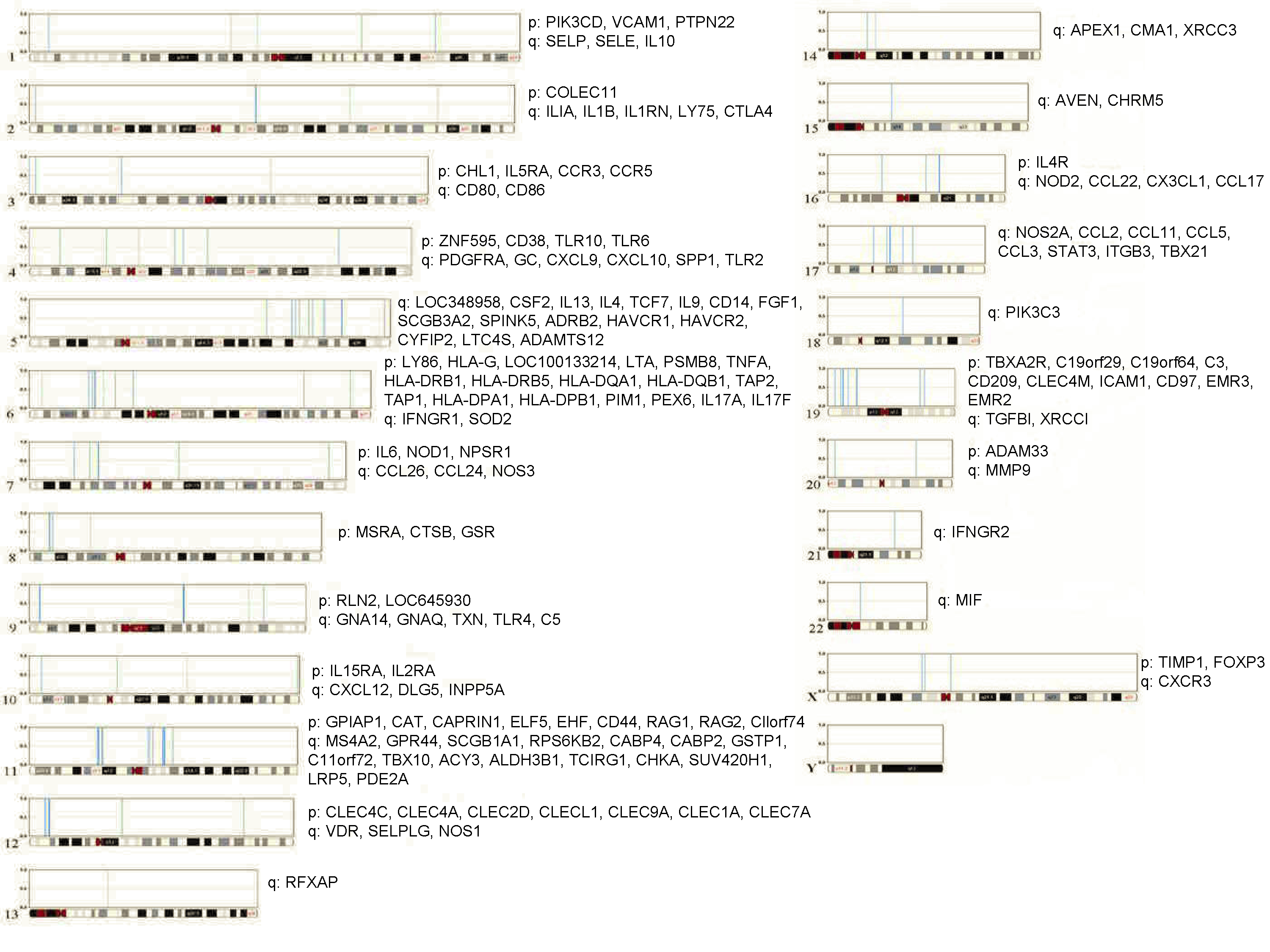

Supplement: Figure S1 — (TIFF) [file pone.0070362.s001.tiff]

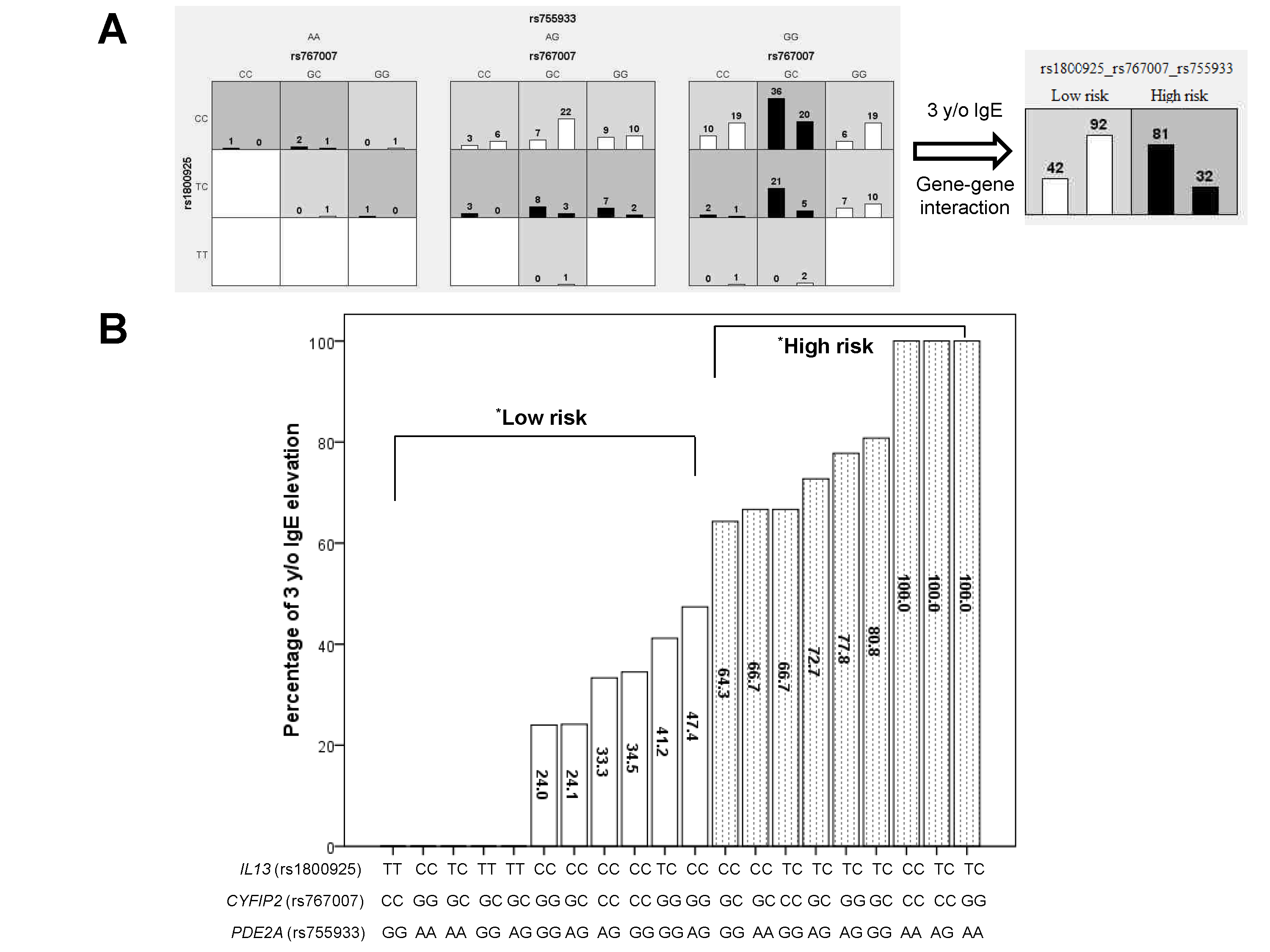

Supplement: Figure S2 — (TIFF) [file pone.0070362.s002.tiff]

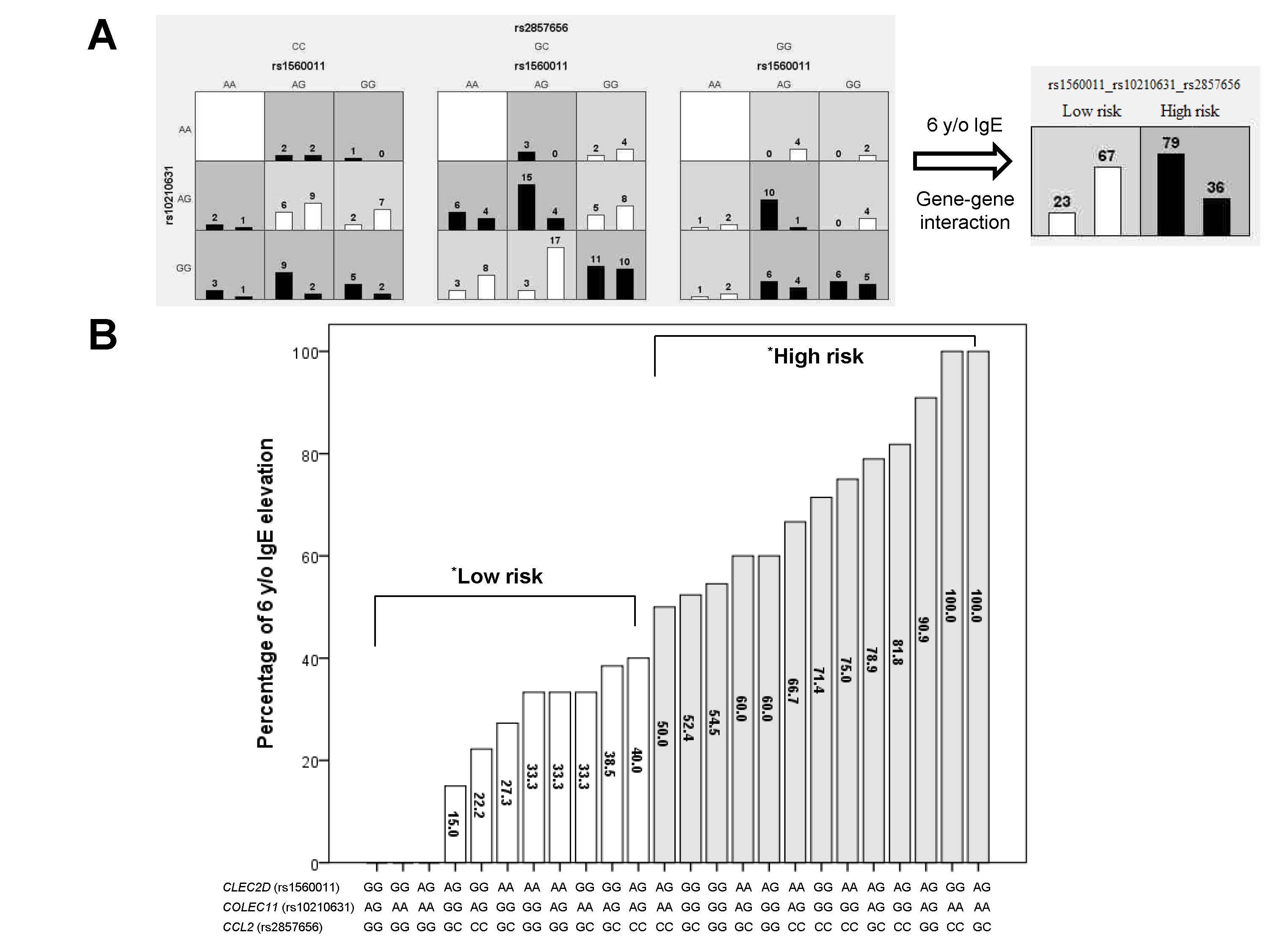

Supplement: Figure S3 — (TIF) [file pone.0070362.s003.tif]

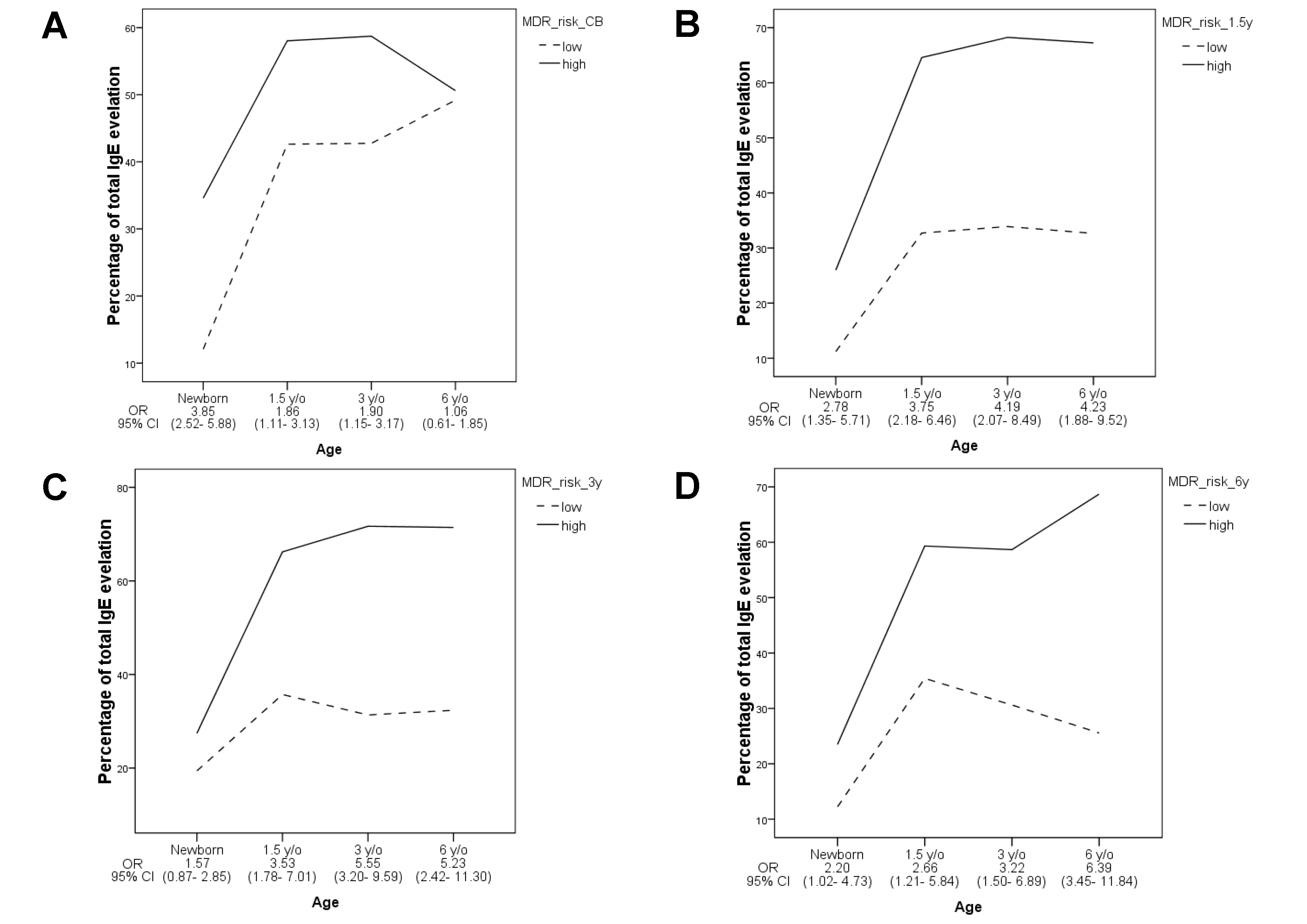

Supplement: Figure S4 — (TIF) [file pone.0070362.s004.tif]
