# Supplementary material for: Different Genetic Associations of the IgE Production among Fetus, Infancy and Childhood
Source: PLoS One. 2013 Aug 1;8(8):e70362. doi: 10.1371/journal.pone.0070362 (PMC3731352; doi:10.1371/journal.pone.0070362)
Supplement: Table S1 — (DOC) [file pone.0070362.s005.doc]

**Table S1A.**

| **Genes** | **dbSNP** | ***P* value** | **OR** | **95% CI** |
| --- | --- | --- | --- | --- |
| Innate immunity | |  |  |  |
| *IL6* | rs1880242 | ns |  |  |
| *CLEC9A* | rs874463 | ns |  |  |
| *CX3CL1* | rs170361 | ns |  |  |
| *CX3CL1* | rs8323 | ns |  |  |
| Adaptive immunity | |  |  |  |
| *IL9* | rs10477776 | ns |  |  |
| *TAP1* | rs2284190 | ns |  |  |
| *RAG2* | rs867801 | ns |  |  |
| *IFNGR2* | rs2268241 | ns |  |  |
| *IFNGR2* | rs9808753 | 0.009 | 0.959 | 0.929-0.990 |
| Stress and response | |  |  |  |
| *FGF1* | rs2282797 | ns |  |  |
| *GNAQ* | rs4744850 | ns |  |  |
| *C11orf74* | rs2422297 | 0.007 | 6.708 | 1.699-26.479 |
| *EHF* | rs717582 | ns |  |  |
| *ADAM33* | rs2280091 | ns |  |  |
| *ADAM33* | rs3918395 | ns |  |  |
| Maternal atopy | | ns |  |  |
| Paternal atopy | | ns |  |  |
| Gender | | ns |  |  |
| Prematurity | | ns |  |  |
| Parental smoking | | ns |  |  |
| CBIgE (≧0.5 kU/L) | | 1.23x10^-4^ | 6.710 | 2.540-17.727 |
| *NPSR1_FGF1* (rs324981_rs2282797) | | 1.46x10^-4^ | 3.246 | 1.768-5.962 |

Notes: Data analyzed are adjustably significant SNPs and demographic data. OR, odds ratio. CI, conﬁdence interval. CBIgE, cold blood IgE. ns, not significant.

**Table S1B.**

| **Genes** | **dbSNP** | ***P* value** | **OR** | **95% CI** |
| --- | --- | --- | --- | --- |
| Innate immunity | |  |  |  |
| *CLEC2D* | rs1560011 | ns |  |  |
| *CX3CL1* | rs170361 | ns |  |  |
| *CD209* | rs4804801 | 0.018 | 1.119 | 1.019-1.229 |
| Adaptive immunity | |  |  |  |
| *IL13* | rs1295686 | ns |  |  |
| *IL13* | rs1800925 | ns |  |  |
| *IL13* | rs20541 | ns |  |  |
| *HAVCR2* | rs11134551 | ns |  |  |
| *TAP1* | rs2284190 | ns |  |  |
| *RAG2* | rs867801 | ns |  |  |
| *IL4R* | rs1801275 | ns |  |  |
| *IFNGR2* | rs2268241 | ns |  |  |
| *IFNGR2* | rs9808753 | ns |  |  |
| *IFNGR2* | rs9976971 | 0.027 | 0.973 | 0.949-0.997 |
| Stress and response | |  |  |  |
| *PDGFRA* | rs4358459 | ns |  |  |
| *GC* | rs4588 | ns |  |  |
| *CYFIP2* | rs767007 | ns |  |  |
| *GSR* | rs8190955 | 0.029 | 0.780 | 0.624-0.975 |
| *C11orf74* | rs2422297 | ns |  |  |
| *GSTP1* | rs1871042 | ns |  |  |
| Maternal atopy | | ns |  |  |
| Paternal atopy | | 0.007 | 2.844 | 1.324-6.113 |
| Gender | | 0.001 | 3.328 | 1.666-6.649 |
| Prematurity | | ns |  |  |
| Parental smoking | | ns |  |  |
| CBIgE (≧0.5 kU/L) | | 0.001 | 4.471 | 1.915-10.440 |
| *IL13_CYFIP2_PDE2A* (rs1800925_rs767007_rs755933) | | 5.98x10^-7^ | 5.713 | 2.882-11.324 |

Notes: Data analyzed are adjustably significant SNPs and demographic data. OR, odds ratio. CI, conﬁdence interval. CBIgE, cold blood IgE. ns, not significant.

**Table S1C.**

| **Genes** | **dbSNP** | ***P* value** | **OR** | **95% CI** |
| --- | --- | --- | --- | --- |
| Innate immunity | |  |  |  |
| *CCR5* | rs1799987 | 0.001 | 1.049 | 1.019-1.080 |
| *TNFA* | rs1800629 | ns |  |  |
| *CD209* | rs7248637 | 3.07x10^-4^ | 1.060 | 1.027-1.094 |
| Adaptive immunity | |  |  |  |
| *IL5RA* | rs163550 | ns |  |  |
| *IL5RA* | rs340833 | ns |  |  |
| *CD80* | rs2629396 | 0.003 | 0.909 | 0.853-0.969 |
| *HAVCR1* | rs953569 | ns |  |  |
| *HLA-DPA1* | rs1431399 | ns |  |  |
| *HLA-DQA1* | rs2040410 | 0.003 | 0.944 | 0909-0.981 |
| Stress and response | |  |  |  |
| *PTPN22* | rs3765598 | ns |  |  |
| *CYFIP2* | rs2863198 | 0.011 | 1.113 | 1.025-1.209 |
| *CYFIP2* | rs3734028 | ns |  |  |
| *CYFIP2* | rs767007 | ns |  |  |
| *EMR3* | rs10410565 | ns |  |  |
| *XRCC1* | rs25487 | ns |  |  |
| Maternal atopy | | 0.010 | 4.019 | 1.393-11.600 |
| Paternal atopy | | 0.050 | 2.462 | 1.001-6.055 |
| Gender | | 1.18x10^-4^ | 6.428 | 2.493-16.574 |
| Prematurity | | ns |  |  |
| Parental smoking | | ns |  |  |
| CBIgE (≧0.5 kU/L) | | 0.042 | 3.392 | 1.043-11.033 |
| *CLEC2D_COLEC11_CCL2* (rs1560011_rs10210631_rs2857656) | | 6.65x10^-7^ | 9.755 | 3.974-23.948 |

Notes: Data analyzed are adjustably significant SNPs and demographic data. OR, odds ratio. CI, conﬁdence interval. CBIgE, cold blood IgE. ns, not significant.
